# How effective are ketamine or esketamine in treatment-resistant depression?

**DOI:** 10.1192/j.eurpsy.2021.886

**Published:** 2021-08-13

**Authors:** N. Veluri, Z. Mansuri

**Affiliations:** 1 N/a, American University of Integrative Sciences, School of Medicine, St.Michale, Barbados; 2 Department Of Psychiatry, Boston Children’s Hospital/Harvard Medical School, Boston, United States of America

**Keywords:** TRD, Ketamine, treatment-resistant depression, esketamine

## Abstract

**Introduction:**

Globally, depression affects millions of individuals. A third of depression patients meet the criteria for treatment-resistant depression (TRD). The N-methyl-D-aspartate receptor antagonist, ketamine, improved depressive symptoms in a span of 24-hours. Recently, the FDA approved esketamine, an enantiomer of ketamine for TRD.

**Objectives:**

To determine the effectiveness of ketamine and esketamine in TRD, and observe their role in suicidality.

**Methods:**

Individual systematic searches were conducted on the PubMed database following the PRISMA protocol (Figure 1). Inclusion criteria included randomized clinical trials (RCT). Search strings were (i) “ketamine” OR “esketamine” AND “treatment-resistant depression” (ii) “ketamine” OR “esketamine” AND “suicide.” Eleven studies were included for depression and five studies for suicidality (Table 1). Comparison analysis for suicide appeared trivial because of only one inclusion eligible esketamine RCT. This review was submitted for registration at PROSPERO. Randomized odds ratios, 95% confidence interval (CI), and heterogeneity were obtained.

**Figure:**
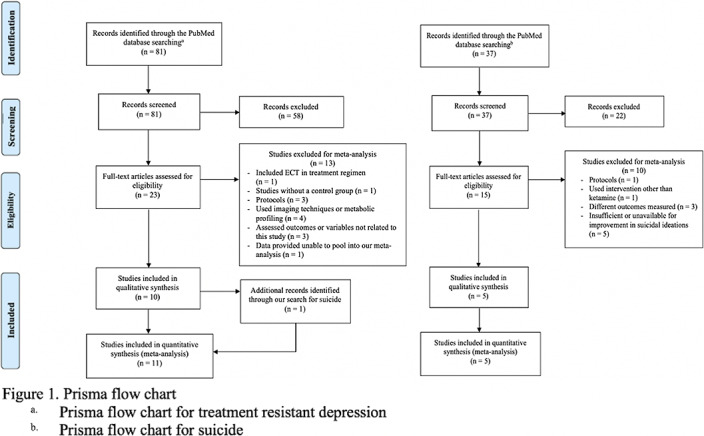

**Results:**

The comprehensive meta-analysis, version 3.0, was used for analysis. Ketamine improved TRD symptoms and reduced suicidality by a nine-fold and three-fold odds, respectively (OR 9.01, CI 4.89–16.6, p<0.001 and OR 2.9, CI 1.67–5.06, p<0.001). Esketamine also improved TRS symptoms (OR= 2.61, 95% CI= 1.56–4.37, p<0.001). The heterogeneity ranged from 11% to 60% between the three groups. Sensitivity analysis did not alter the results.

**Figure:**
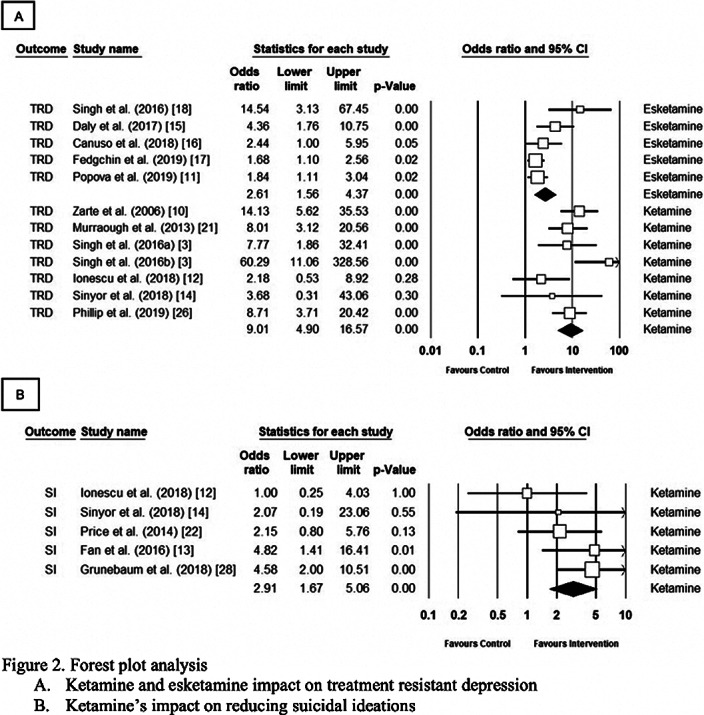

**Figure:**
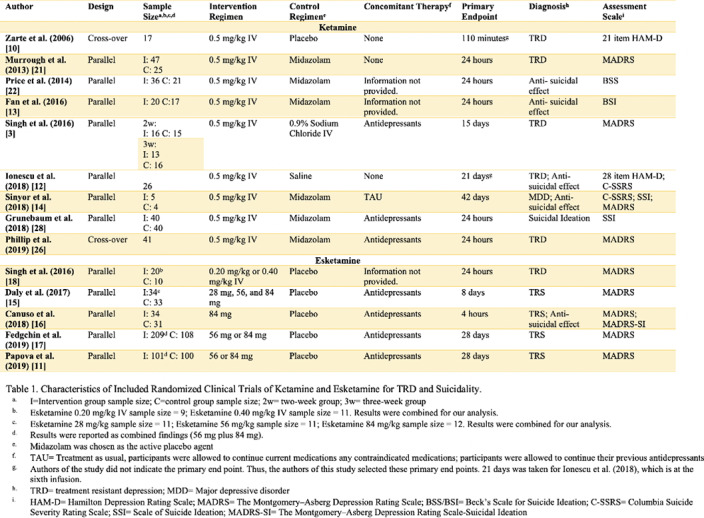

**Conclusions:**

Findings must be cautiously interpreted as the primary endpoint differed. The primary endpoint was set at 24-hours and 28-days for ketamine and esketamine, respectively. Esketamine’s effectiveness over 28 days appears promising for TRD. Current aim should consist of structured guidance for clinicians in esketamine administration.

